# Modeling Adolescent Social Inclusion to Improve School Completion

**DOI:** 10.1007/s10964-023-01792-9

**Published:** 2023-05-29

**Authors:** Heidi M. Renner, Bosco Rowland, Delyse Hutchinson, John W. Toumbourou

**Affiliations:** 1 Centre for Social and Early Emotional Development (SEED), School of Psychology, Faculty of Health, Deakin University, Geelong, VIC Australia; 2 Murdoch Children’s Research Institute, Melbourne Children’s LifeCourse Initiative, Parkville, VIC Australia; 3 Eastern Health Clinical School & Monash Addiction Research Centre, Monash University, Richmond, VIC Australia; 4 Murdoch Children’s Research Institute, Centre for Adolescent Health, Melbourne, VIC Australia; 5 Department of Paediatrics, Royal Children’s Hospital, University of Melbourne, Melbourne, VIC Australia; 6 National Drug and Alcohol Research Centre, Faculty of Medicine, University of New South Wales, Sydney, NSW Australia

**Keywords:** Social inclusion, Adolescence, School completion, Equity, Social identity, Belonging

## Abstract

Enhancing social inclusion in young people could increase engagement in education, yet few longitudinal studies have examined this relationship. This study aimed to identify whether social inclusion in an Australian adolescent sample predicted high school completion three years later. Using state-representative data from the International Youth Development Study, two waves of the youngest cohort (51.6% female and 94.6% Australian born) during mid-adolescence (*n* = 825, *M*_*age*_ = 15.99, *SD* = 0.39) and post-secondary school (*n* = 809, *M*_*age*_ = 19.03, *SD* = 0.44) were analyzed. Factor analysis identified a 4-factor structure that represented an overarching social inclusion construct: (1) Citizenship, (2) Connectedness to Community, (3) Connectedness to Family, and (4) Connectedness to and Participation in School. Multivariate regression analyses indicated higher social inclusion levels in mid-adolescence predicted an increased likelihood of high school completion three years later. The implementation of strategies that incorporate the enhancement of social inclusion may improve educational outcomes for young people.

## Introduction

Inclusion in society conveys social, psychological, and economic benefits to individuals and to society more broadly (Filia et al., 2018; Shepherd & Parsonage, 2011). Social inclusion can act as a protective mechanism that can help lessen problems such as racism, bullying, and ostracism, and the impact these have on health, social, and emotional outcomes for adolescents (Floyd et al., 2017; Thomas & Griffin, 2021). Promoting and cultivating social inclusion during the adolescent years may also redress educational inequity and provide young people a strong foundation for a variety of positive outcomes, such as school completion. There have been various attempts to operationalize the assessment of social inclusion (Cordier et al., 2017); however, prior research has predominantly focused on examining proxies for social inclusion in relation to educational outcomes, which are often restricted to one societal level. The current study aimed to identify a multidimensional measure of social inclusion and examine the predictive relationship with school completion in a longitudinal sample of Australian adolescents.

### Conceptualizing and Assessing Social Inclusion

Social inclusion can be conceptualized in various ways. The key features usually include constructs of equity and social justice and tend to focus on human potential rather than deficiency (Gidley et al., 2010). The experience of belonging to, and being included in a group, as well as having access to resources and opportunities (e.g., social capital), are core elements of social inclusion (Abrams et al., 2005). Despite varied conceptualizations, three key domains are evident in the literature: (1) “participation” represents involvement in societal activities (e.g., education, employment, recreation) and is often contingent on *access* to resources (Shepherd & Parsonage, 2011); (2) “connectedness and a sense of belonging” arises through *participation* in society and represents the experience of involvement in social networks (e.g., in school, with family, as part of a sporting team); and (3) “citizenship and rights” reflects the *civic* aspect of involvement through established systems, and represents political or altruistic interests of the individual, and societal rights and obligations (Cordier et al., 2017; Gidley et al., 2010). Conceptualizations of social inclusion for children and adolescents sometimes tap into two additional domains: (4) “access” refers to the ability to obtain resources and opportunities, often enabled through social capital, and (5) “empowerment” encompasses human potential and a sense of agency to influence one’s environment and be heard by others in society (Gidley et al., 2010; Rose et al., 2012).

Several existing measurement tools seek to capture social inclusion; however, contrary to multidimensional conceptualizations of social inclusion evident in the literature, most scales tend to focus predominantly on measurement in one or two key domains only (Cordier et al., 2017; Magson et al., 2014; Slaten et al., 2019). This approach does not adequately incorporate the reported multidimensionality of the construct. Most measurement tools are designed for adults, and often for use in clinical populations (Cordier et al., 2017), however more recently, measurement scales for a child and adolescent population have been developed (Magson et al., 2014; Moyano et al., 2020; Slaten et al., 2019; Zurbriggen et al., 2019). Some of these measures take a multidimensional and bioecological approach by including dimensions across a variety of levels. These levels include factors associated with the individual, peers, family, school, and community or neighborhood.

### Social Inclusion and Adolescent Educational Outcomes

Evidence indicates individual aspects of social inclusion may have a positive impact on adolescent school completion, through access to opportunities (Perreira et al., 2006; Ryabov, 2011; Wu et al., 2014). Parental involvement, school-level inclusion, and extra-curricular activities (such as sports or performing arts), have been shown to decrease school dropout and increase school completion (Cemalcilar & Gökşen, 2014; Hughes et al., 2016). For high-risk students, social-emotional skills training, mentoring, and community service were found to improve high school completion (Hahn et al., 2015).

Other factors associated with secondary school completion, include: (a) demographics (e.g., low parent education), (b) ethnicity, (c) behavioral and educational problems in school, (d) substance use, (e) antisocial behavior, (f) childhood emotional and behavioral problems, (g) parenting problems, and (h) mental health problems (Goldfeld et al., 2018; Gubbels et al., 2019; Weber et al., 2018). Some of these factors are difficult to influence; therefore, exploring modifiable factors that improve educational outcomes for all Australian youth, such as social inclusion, is an imperative. The Communities That Care youth survey is increasingly used to monitor socioecological variables that affect adolescent health (Thurow et al., 2021). Using these surveys to monitor adolescent experiences of inclusion in society may offer opportunities to improve protective mechanisms that can reduce problems such as racism, bullying, and educational disengagement.

## The Current Study

Most adolescent social inclusion measurement tools capture inclusion across one or two key domains, while research often uses individual aspects of social inclusion as proxies, rather than using a comprehensive, multidimensional measure when examining school completion. Given the limits of previous research, the current study aimed to identify a group of variables that reasonably operationalized and measured the latent variable of adolescent social inclusion across several bioecological levels and within a sample of Australian youth. In addition, the study aimed to examine the extent to which social inclusion during adolescence predicted Year 12 completion in young adulthood, while adjusting for covariates (gender, ethnicity, substance use, antisocial behavior, childhood emotional and behavioral problems, parenting problems, and mental health problems). Based on previous literature, it was hypothesized that social inclusion in adolescence would comprise five domains, namely: participation, connectedness and belonging, citizenship and rights, access, and empowerment. Also consistent with previous literature, it was hypothesized that social inclusion in adolescence would predict Year 12 completion, with higher rates of school completion among those reporting greater social inclusion.

## Methods

### Participants

This study used data from a prospective version of the Communities That Care youth survey known as the International Youth Development Study (IYDS). The IYDS is a cross-national longitudinal study that commenced in 2002 in Victoria, Australia and Washington State, the United States of America (McMorris et al., 2007). The IYDS investigated adolescent risk and protective factors and developmental outcomes, across multiple bioecological levels, with a particular focus on substance use and delinquency. Using a two-stage cluster sampling approach, at both the suburb and school level, a state representative sample of students across grades 5, 7, and 9 (youngest, middle, and oldest respectively) were recruited. In Australia, ethics approval was granted for the IYDS by the Royal Children’s Hospital Ethics in Human Research Committee; extensive detail on the IYDS design and sampling methodology has been presented previously (McMorris et al., 2007).

The current study focused on the youngest cohort (recruited in Grade 5) from the Australian sample. Data from wave 5 in 2007 (*n* = 825; average age of 16 years) when participants were in Year 10, the last year of compulsory schooling in Australia, and wave 7 in 2010 (*n* = 809; average age of 19 years), when all participants were post-secondary education, were used in the current study. Informed consent was provided by both parents and participants in wave 5, and by participants in wave 7 (McMorris et al., 2007). Wave 5 data was collected via youth self-report surveys delivered during class time at school, while wave 7 self-report surveys were completed by participants via either paper or online survey or via telephone interview. Surveys were Australian adaptations of the Communities that Care youth survey. Table 1 outlines sample frequencies and key demographics of the participants.

**Table 1:** Sample size, distribution, retention rates, and key demographics for the Australian youngest cohort of the IYDS

Wave	Year	*n*	Age	
			M	SD
Wave 1 (baseline)	2002	927	10.98	0.40
Wave 5	2007	825	15.99	0.39
Wave 7	2010	809	19.03	0.44
Missing^a^		16		

Demographics @ wave 5		*n*	%	
Gender		825		
Male		399	48	
Female		426	52	
Country of birth		789		
Australia		746	95	
Country other than Australia		43	5	
Aboriginal or Torres Strait Islander		12	0.01	
Household income by quintiles^b^		696		
Less than $30,000 (lowest quintile)		165	24	
Between $30,001 and $50,000 (second)		146	21	
Between $50,001 and $70,000 (third)		158	23	
Between $70,001 and $90,000 (fourth)		126	18	
Over $90,000 (highest quintile)		101	15	
Retention rate between wave 1 and wave 5			89	
Retention rate between wave 5 and 7			98	

^a^Missing represents participants lost to follow-up between wave 5 and 7

^b^Household income is in Australian currency (AUD)

### Measures

#### Social inclusion indicators

Social inclusion indicator selection was guided by a systematic review of social inclusion measurement tools and scales (Cordier et al., 2017), along with additional youth scales identified after the review (Moyano et al., 2020; Zurbriggen et al., 2019). Five key domains across four bioecological levels (individual/peer, family, school, and community) were theorized from the adolescent measurement tools: (1) participation, (2) connectedness and belonging, (3) citizenship and rights, (4) access, and (5) empowerment.

Items within the IYDS that were exemplars of these domains were selected to ensure theoretical consistency and formed a core group of 32 indicators (see Appendices A and B in the supplementary material). Analysis of a correlation matrix of the indicators resulted in a final selection of 21 categorical and continuous indicators (see Table 2) across the relevant bioecological levels. Pearson correlations of the 21 indicators included in the final analyses ranged between *r* = .02 and *r* = .83 (see Appendix C).

**Table 2:** Descriptive statistics for social inclusion indicators at wave 5 (*N* = 825) and school completion at wave 7 (*N* = 809)

Indicator (wave 5)	*N*	M	SD	Range
Individual - Peer Level				
1. Been a leader in group or organization	818	1.92	1.22	1–5
2. Made a personal effort to care for environment	823	4.06	2.31	1–8
3. Helped someone feel better when upset	823	4.83	2.03	1–8
4. Helped student with difficulty at school	820	3.88	1.99	1–8
5. Prosocial behavior^a^	822	4.39	1.98	1–8
6. Civic engagement^a^	818	1.69	0.79	1–5
Family Level				
7. Parents notice when child doing good job	820	3.05	0.86	1–4
8. Parents tell child when they are proud of them	818	2.95	0.89	1–4
9. Family attachment^a^	815	2.98	0.70	1–4
10. Family opportunities for prosocial involvement^a^	820	3.10	0.66	1–4
School Level				
11. Lots of chances to be part of class discussion	808	3.26	0.64	1–4
12. Feel safe at school	806	3.25	0.69	1–4
13. School climate - belonging/acceptance^a^	805	2.98	0.52	1–4
14. School climate - student participation^a^	806	3.01	0.53	1–4
15. School climate - supportive teacher relationships^a^	805	2.80	0.54	1–4
16. Commitment to school^a^	809	3.80	0.59	1–5
Community Level				
17. Lots of adults to talk to in neighborhood	818	2.25	0.96	1–4
18. Kids empowered in neighborhood (make decisions)	815	2.05	0.89	1–4
19. Adults pay attention to kids in neighborhood	815	2.18	0.93	1–4
20. Neighborhood attachment^a^	817	3.09	0.72	1–4
21. Community rewards for prosocial involvement^a^	819	2.16	0.88	1–4
Educational outcome (wave 7)				
Completed Year 12 by wave 7	797	0.76	0.43	
No	194			
Yes	603			
Missing	12			

^a^Continuous variable

#### School completion

In Australia, school education is compulsory until the age of 16 or 17 years, or at completion of Year 10, varying by state and territory, with Year 12 the final year of secondary school prior to tertiary education. Educational measures and schooling structure vary internationally; thus, to ensure the outcome measure was meaningful in Australia, the Australian Bureau of Statistics (ABS) response options for secondary school completion were used and then dichotomized (Australian Bureau of Statistics, 2009). Specifically, self-reported highest year level of secondary school completed was dichotomized: (a) completed Year 12 (or equivalent) or, (b) did not complete Year 12.

#### Covariates

Significant independent covariates across seven areas: (a) demographics, (b) ethnicity, (c) substance use, (d) antisocial behavior, (e) childhood emotional and behavioral problems, (f) parenting problems, and (g) mental health problems (Goldfeld et al., 2018; Gubbels et al., 2019; Weber et al., 2018) were included in the analysis to control for potential confounding effects (see Appendix D for specifics on the covariates). Prior academic achievement was not included as a covariate as it was theorized to be on the causal pathway between social inclusion and school completion (van Stralen et al., 2010).

### Analytic Approach

The analysis involved four steps. First, 32 indicators that aligned with the social inclusion domains identified in the literature (participation, connectedness and belonging, citizenship, access, and empowerment) were selected. These indicators were recoded to ensure directional uniformity, with increasing values indicating increasing positive behaviors. A correlation matrix was performed using StataBE version 17.0 (StataCorp, 2021) to examine underlying relationships between indicators; indicators with few moderate correlations were removed (“moderate” defined as *r* > 0.30). Tests to ensure suitability for exploratory factor analysis, multicollinearity, and multivariate normality were conducted; the determinant of the correlation matrix, Bartlett test of sphericity, and Kaiser-Meyer-Olkin measure of sampling adequacy were calculated (Watkins, 2021).

Second, using Mplus Version 8.8 (Muthén & Muthén, 1998–2022), a series of exploratory factor analyses (EFA) (2- to 7-factor) were conducted to explore relationships between the indicators and alignment with theoretical conceptualizations of social inclusion. Mplus uses full information maximum likelihood (FIML) parameter estimation to account for missing data and has the capacity to incorporate both categorical and continuous variables in analyses. The model fit statistics, interpretability, and meaningfulness were assessed to determine the best fitting model; comparative fit index (CFI), Tucker-Lewis index (TLI), standardized root mean square residual (SRMR), and root mean square error of approximation (RMSEA) were used.

Third, confirmatory factor analyses (CFA) were conducted in Mplus to test the structure of social inclusion proposed by the EFA and confirm the indicators sufficiently reflected the domains of the underlying construct (Hair et al., 2014). Interpretability of the factor structure informed the selection of the final model. A second order CFA was then conducted to test the theoretical higher order construct of social inclusion, and factor scores were saved for use in regression analyses. The same fit indices for the EFA were used to assess CFA model fit.

Finally, logistic regression analyses were conducted in StataBE version 17.0 (StataCorp, 2021) using a confirmatory specification approach, whereby all variables were entered into the model together to identify whether experienced level of social inclusion in Year 10 (wave 5, age 16) predicted completion of Year 12 (wave 7, age 19), with and without adjusting for significant covariates (Hair et al., 2014). Clustering effects by school were accounted for using the ‘svyset’ command and ‘svy’ prefix in the regression analysis, and sensitivity analyses were conducted to test patterns of missing data. A multiple imputation analysis (*K* = 10) was undertaken to examine inflation of the results for the regression model, due to missing data.

## Results

### Sample Characteristics

The sample was approximately evenly spread across the two genders measured (male, 48%; female, 52%), was mostly Australian born (95%), and was normally distributed across quintiles of household income: the lowest quintile reported less than $30,000 per annum (24%), the middle three quintiles reported between $30,000 and $90,000 per annum (62%), and the highest quintile reported between $90,000 to over $200,000 per annum (15%) (see Table 1). The IYDS retention rate for the youngest cohort was high with 89% participants retained between baseline and wave 5, and 98% of wave 5 participants retained in the wave 7 sample.

### Social Inclusion Indicator Selection

From an initial sample of 32 indicators (see supplementary material), 11 indicators with low correlations were excluded from further analyses; three indicators representing “empowerment”, two representing “access”, and six distributed across the other three domains were excluded, resulting in a final group of 21 indicators. As seen in Table 2, the youngest cohort exhibited mostly positive behaviors across the indicators at the individual/peer, family, school, and community levels. In addition, most participants had completed Year 12 by wave 7 (76%). The continuous indicators were normally distributed with low levels of skew and kurtosis (skew < ±2.0 and kurtosis < ±7.0) (Watkins, 2021). The educational outcome was skewed towards school completion, as expected.

### Exploratory Modeling of Social Inclusion

The correlation matrix, Kaiser-Meyer-Olkin Measure of Sampling Adequacy (*KMO* = 0.87) and a significant Bartlett’s test of sphericity (*p* < 0.001) confirmed suitability for an EFA. Model fit statistics indicated the 6-factor model was the best fitting model; however, the most theoretically meaningful, interpretable, and parsimonious model was the 4-factor model, and therefore was the model retained (CFI/TLI = 0.95/0.92; SRMR = 0.04; RMSEA = 0.07) (see Appendix E). In addition, the 4-factor model had the optimum number of items per factor (4–6) (Watkins, 2021), whereas the 6-factor model allocated only one indicator (family attachment) to the sixth factor, which is insufficient (see Appendix F).

The 4-factor model was deemed to represent four factors: (1) Citizenship, (2) Connectedness to Community, (3) Connectedness to Family, and (4) Connectedness to and Participation in School, across the individual/peer, community, family, and school levels, respectively. For simplicity, the concept of “connectedness and a sense of belonging” was shortened to “connectedness”, e.g., Connectedness to Family.

### Confirmatory Modeling of Social Inclusion

Following exploratory analyses, a 4-factor CFA was conducted to ensure the theoretical structure identified in the EFA was supported. Model fit statistics indicated adequate fit, and factor loadings showed the indicators were good measures of the underlying constructs (*R* = 0.54–0.93); however, communalities varied across the indicators, from poor (*R*^*2*^ = 0.29) to good (*R*^*2*^ = 0.87). The factor covariance matrix showed low to moderate correlations (*r* = 0.25–0.45), indicating good discriminant validity, while the factors were sufficiently correlated to reflect an overarching construct (see Table 3).

**Table 3:** Factor loadings, squared loadings, and model fit statistics for 4-factor CFA

Indicator	Factor loading				*R*^*2*^
	1	2	3	4	
Individual - Peer Level					
1. Helped student with difficulty at school	0.80				0.64
2. Helped someone feel better when upset	0.75				0.56
3. Prosocial behavior^a^	0.75				0.56
4. Made a personal effort to care for environment	0.64				0.42
5. Been leader in group or organization	0.56				0.31
6. Civic engagement^a^	0.56				0.31
Community Level					
7. Kids listened to in neighborhood		0.93			0.87
8. Kids empowered in neighborhood		0.88			0.77
9. Community rewards for prosocial behavior		0.86			0.74
10. Lots of adults to talk to in neighborhood		0.80			0.63
11. Neighborhood attachment^a^		0.54			0.29
Family Level					
12. Parents notice when child doing good job			0.92		0.85
13. Parents tell child when they are proud of them			0.87		0.76
14. Family opportunities for prosocial involvement			0.80		0.64
15. Family attachment^a^			0.68		0.46
School Level					
16. School climate - supportive teacher relationships^a^				0.80	0.64
17. School climate - student participation^a^				0.73	0.53
18. School climate - belonging/acceptance^a^				0.72	0.52
19. Lots of chances to be part of class discussion				0.66	0.44
20. Commitment to school^a^				0.65	0.42
21. Feel safe at school				0.64	0.40
Cronbach’s alpha (α)	0.80	0.87	0.86	0.83	
Covariance matrix (*r*)					
1. Citizenship					
2. Connectedness to Community	0.25***				
3. Connectedness to Family	0.25***	0.41***			
4. Connectedness to and Participation in School	0.45***	0.42***	0.44***		
Model fit				Estimate	
Comparative fit index (CFI)				0.91	
Tucker-Lewis index (TLI)				0.90	
Standardized root mean square residual (SRMR)				0.06	
Root mean square error of approximation (RMSEA)				0.08	

Factors 1, 2, 3, and 4 refer to (1) Citizenship, (2) Connectedness to Community, (3) Connectedness to Family, and (4) Connectedness to and Participation in School, respectively. Factor loadings are standardized

^a^Continuous variable. *R*^*2*^ = squared standardized loading. *r* = Pearson’s correlation coefficient

***p < 0.001

A second order examination of the four domains identified a good model fit, indicating support for the four subdomains, and reflecting an overall social inclusion construct (see Table 4). All factor loadings were adequate (*R* = 0.50–0.78); however, communalities ranged from poor to good (*R*^*2*^ = 0.25–0.60), indicating between 25–60% of the total variance was shared between variables (Hair et al., 2014). A social inclusion factor score was calculated for each respondent. Cronbach’s alpha for the social inclusion factor (α = 0.75) indicated the measure had acceptable reliability (Hair et al., 2014). Cronbach’s alpha for each of the four factors that made up social inclusion ranged from 0.80 to 0.87, indicating overall acceptable reliability for this measure.

**Table 4:** Factor loadings, squared loadings, and model fit statistics for social inclusion second Order CFA

Factor	Factor loading	*R*^*2*^
1. Citizenship	0.50	0.25
2. Connectedness to Community	0.57	0.32
3. Connectedness to Family	0.60	0.36
4. Connectedness to and Participation in School	0.78	0.60
Cronbach’s alpha (α)	0.75	
Model fit		Estimate
Comparative fit index (CFI)		0.92
Tucker-Lewis index (TLI)		0.91
Standardized root mean square residual (SRMR)		0.07
Root mean square error of approximation (RMSEA)		0.08

Factor loadings are standardized

*R*^*2*^ = squared standardized loading

**Fig. 1:**
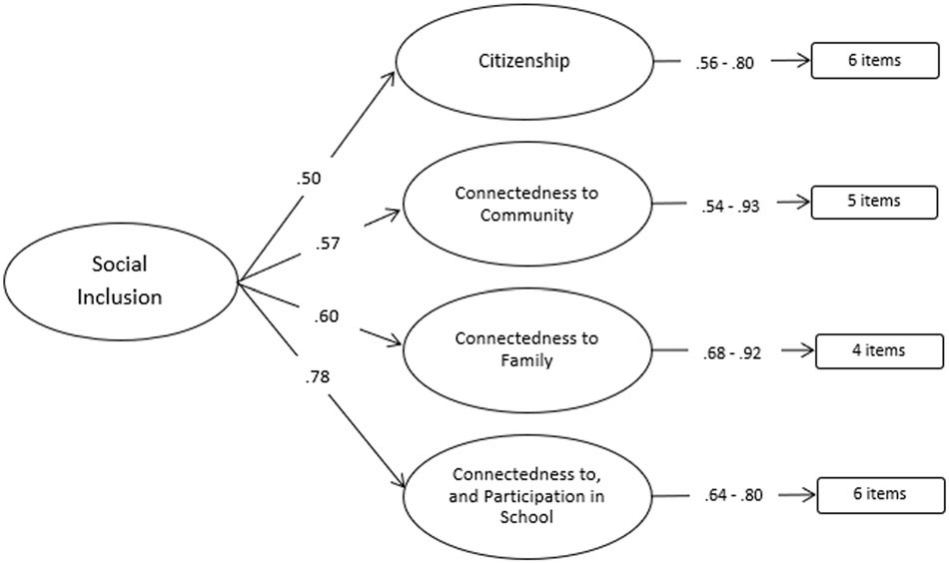
Second Order Confirmatory Factor Analysis of Social Inclusion. Diagrammatic representation of the second order CFA of social inclusion, including standardized factor loadings for the four factors and the factor loading range for the indicators

Figure 1 shows the relationships between the factors for the latent variable social inclusion.

### Social Inclusion Prediction of School Completion

The social inclusion factor scores were divided into quartiles and converted to an ordinal categorical variable, as the continuous variable when used in the regression model resulted in very wide confidence intervals (OR = 2.89, 95%CI [1.07, 7.81], *p* = 0.036). In addition, this served to improve interpretability and applicability for use by educators and policy makers. The cut points for the quartiles were: (1) −1.203 to −0.153 (*n* = 207), (2) −0.152 to −0.001 (*n* = 208), (3) 0 to 0.167 (*n* = 204), and (4) 0.168 to 0.738 (*n* = 206). Table 5 presents school completion frequencies and percentages for the social inclusion quartiles. Overall, 78% of the wave 7 sample completed Year 12, or equivalent, by 2010, with this percentage varying according to the level of social inclusion, from the lowest (first quartile; 67%) to the highest level (fourth quartile; 87%).

**Table 5:** Frequencies for Wave 7 Sample (2010; *N* = 809), Year 12 School Completion by Predicted Level of Social Inclusion (Unadjusted for Confounders)

Social inclusion level by quartile	*n*	Year 12 school completion			
		Did not complete year 12 (or equivalent)		Completed year 12 (or equivalent)	
		*n*	%	*n*	%
First quartile (lowest level)	175	58	33.1	117	66.9
Second quartile	188	45	23.9	143	76.1
Third quartile	185	32	17.3	153	82.7
Fourth quartile (highest level)	185	24	13.0	161	87.0
Totals	733	159	21.7	574	78.3
Missing^a^	76				

^a^Missing value is calculated from the difference between wave 7 and the final sample used in the analysis. Within the overall missing value, 12 were wave 7 participants who did not respond to the school completion items (non-respondents) and 64 were missing from wave 5 but participated in wave 7

Table 6 shows the results of the unadjusted and adjusted logistic regression analyses predicting Year 12 school completion from adolescent social inclusion. Adjusting for covariates, higher levels of social inclusion predicted a higher likelihood of Year 12 school completion, when compared to the lowest level of social inclusion. Participants with the highest social inclusion levels (fourth quartile) were 2.72 times (95% CI [1.40, 5.28], *p* = 0.003) more likely to complete Year 12 than those with the lowest social inclusion levels (first quartile). In addition, those with moderately high social inclusion (third quartile) were also more likely to complete Year 12 (*OR* = 2.23, 95% CI [1.25, 3.97], *p* = 0.007) compared to those with the lowest level of social inclusion.

**Table 6:** Unadjusted and adjusted logistic regression models predicting school completion (Year 12 Completion) from social inclusion second order factor, controlling for significant covariates

Variable	Non-imputed			Imputed		
	OR	95% CI		OR	95% CI	
		LL	UL		LL	UL
Unadjusted model						
Social inclusion						
First quartile (referent)	N/A	N/A	N/A	N/A	N/A	N/A
Second quartile	1.58	1.00	2.49	1.41	0.90	2.21
Third quartile	2.37***	1.50	3.75	2.09**	1.29	3.40
Fourth quartile (highest 25%)	3.33***	1.95	5.68	2.95***	1.75	4.98
Adjusted model						
Social inclusion						
First quartile (referent)	N/A	N/A	N/A	N/A	N/A	N/A
Second quartile	1.45	0.86	2.42	1.37	0.84	2.23
Third quartile	2.23**	1.25	3.97	1.95*	1.14	3.35
Fourth quartile (highest 25%)	2.72**	1.40	5.28	2.57**	1.41	4.68
Significant Covariates						
Gender—female	1.37	0.90	2.10	1.18	0.80	1.74
Parental ethnic background – non-Australian	2.70**	1.42	5.16	1.97**	1.28	3.01
Current alcohol use (past 30 days)	0.48**	0.29	0.79	0.53**	0.34	0.84
Perceived availability of drugs in community	1.08	0.82	1.40	1.12	0.87	1.44
Perceived rewards for antisocial involvement	1.21	0.88	1.66	1.14	0.91	1.43
Parental attitudes favorable toward antisocial behavior	0.84	0.49	1.45	0.82	0.56	1.20
Childhood behavior problems, concentration/attention	0.69**	0.53	0.90	0.74*	0.57	0.97
Sensation seeking	0.95	0.81	1.11	0.95	0.82	1.11
Parental overcontrol	1.29	0.99	1.67	1.31*	1.03	1.66
Depression symptomology	1.16	0.77	1.77	1.08	0.72	1.62

First quartile (lowest 25%) is the reference group

*OR* odds ratio, *CI* confidence interval, *LL* lower limit, *UL* upper limit

*p < 0.05; **p < 0.01; ***p < 0.001

Parental non-Australian background, current alcohol use (past 30 days), and childhood behavior problems (concentration/attention) were significant covariates in the model (*OR* = 2.70, 95% CI [1.42, 5.16], *p* = 0.002; *OR* = 0.48, 95% CI [0.29, 0.79], *p* = 0.004; and *OR* = 0.69, 95% CI [0.53, 0.90], *p* = 0.007, respectively); however, the other covariates did not reach significance.

Logistic regression results using imputed values identified a significant but slightly smaller odds ratio for the third and fourth level of social inclusion (*OR* = 1.95, 95% CI [1.14, 3.35], *p* = 0.015; *OR* = 2.57, 95% CI [1.41, 4.68], *p* = 0.002, respectively) (see Table 6). Further, sensitivity analyses demonstrated level of social inclusion did not predict missingness while controlling for other variables in the model (*OR* = 0.75, 95% CI [0.36, 1.56], *p* = 0.446 and *OR* = 1.07, 95% CI [0.51, 2.25], *p* = 0.856, respectively; see Appendix G in supplementary material).

## Discussion

Inclusion in society is beneficial to physical and mental health and social and emotional outcomes for adolescents. While boosting social inclusion has the potential to improve educational trajectories, few longitudinal studies have examined this relationship using a multidimensional measure of social inclusion for this complex construct. This study aimed to identify and measure social inclusion and test the prospective association with subsequent school completion, in a longitudinal cohort of Australian youth. The hypothesis that social inclusion would be represented by five domains reflecting participation, connectedness and belonging, citizenship and rights, access, and empowerment was not supported. The hypothesis that Year 12 completion would increase as a function of the level of social inclusion during adolescence was supported. These findings have important implications for how social inclusion is understood in policy and practice, and how social inclusion can be used to promote school completion.

### Modeling Social Inclusion

This study found social inclusion was operationalized by four factors: (1) Citizenship, (2) Connectedness to Community, (3) Connectedness to Family, and (4) Connectedness to and Participation in School. Two of the five key domains hypothesized were represented, “citizenship and rights” and “connectedness and belonging” and were spread across a number of levels: individual, community, family, and school. Notably, existing adolescent scales also often measure across multiple levels and tend to focus on one or two social inclusion domains (Magson et al., 2014; Slaten et al., 2019).

Engaging in groups at different levels of society is part of the process of establishing and developing self-concept and identity (Dovidio et al., 2005), which subsequently influences an individual’s experience of inclusion. This is especially prominent for adolescents as most of their time is spent engaging in discrete groups based on societal expectations; most weekdays are spent at school and with peers, while the balance is spent with family or engaging in extra-curricular activities and employment. Thus, adolescent social inclusion measures should aim to represent multiple domains.

“Participation”, “access”, and “empowerment” were not represented in the factor structure. However, some aspects of both “participation” and “access” were represented within the Connectedness (family, school, and community) and Citizenship domains due to conceptual overlap. For example, early childhood experiences and environment enable *access* to resources and opportunities which leads to increased prosocial behavior (Citizenship) (Twenge & Baumeister, 2005), and through *participation* in society and group membership, *connections* with others are developed and reinforced. In addition, a school specific “participation” indicator included in the third factor/domain (Connectedness to and Participation in School), highlights the interconnection between participation and connection. Given the structured nature of educational institutions, and the monitoring of involvement and lack of involvement (e.g., school attendance, suspensions), perhaps this is not so surprising.

While “participation” and “access” were partly incorporated into the four social inclusion factors, “empowerment” was not evident, despite the literature suggesting this is an important aspect of social inclusion for adolescents. Lack of representation in the factor structure may be partly explained by an insufficient number of indicators in the data analysis, but as “empowerment” is a more abstract domain, it is also more difficult to measure. Inclusion of indicators that specifically represent individual agency, demonstrate a youth voice, and demonstrate decision making that is entrusted to adolescents may better capture this key domain.

While it is important to get a clear picture of each of the domains that make up social inclusion, this study primarily sought to measure the overarching social inclusion construct. The social inclusion factor provided some explanation for the variance in each of the four lower order factors; connection to school and family contributed most strongly to a broader sense of societal inclusion for adolescents (*R*^2^ = 0.60 and 0.36, respectively), and Connectedness to Community, and Citizenship explained the smallest amount of variance (*R*^*2*^ = 0.32 and 0.25, respectively). A vast amount of a child’s life is spent at school and home, compared to volunteering and community level activities; hence social inclusion more broadly is likely to be strongly represented by school and family level inclusion.

Social identity and group membership may be more dependent on belonging at school and amongst peers than helping or prosocial behaviors in the community (Bottrell & Goodwin, 2011). The current study conceptualized social inclusion as the experience of belonging and being included in a group, and the findings supported this through strong representation across “connectedness and belonging”. Although this study did not find support for three of the five key domains identified in the literature, it does suggest a multidimensional measure with a minimum of two domains.

### Prediction of School Completion

Consistent with the hypothesis, social inclusion was found to predict secondary school completion. Social inclusion in adolescence at age 16 was predictive of secondary school (Year 12) completion at 19 years of age. Specifically, odds of completing Year 12 were 2.72 times higher for adolescents with the highest level of social inclusion, compared to those with the lowest level of social inclusion. Variables such as mental health and antisocial behavior did not significantly predict school completion. While this is inconsistent with some previous research (Gubbels et al., 2019), this could be due to the large number of control variables in the model and the overlapping variance between these variables.

Overall, social inclusion provides multiple benefits to adolescents. It can do this through group membership at the individual/peer, family, school, and community levels, such as access to resources, to group identity, and to increased self-esteem (Emler & Reicher, 2005), which may in turn increase academic success and subsequent school completion (Klimstra & van Doeselaar, 2017). In contrast, poor social inclusion in school is likely to make it difficult to participate and engage in education, irrespective of one’s ability (Emler & Reicher, 2005), and may forecast environments where racism, bullying, and exclusion are normalised. Identifying as part of an excluded group results in alignment with the group’s social norms, which at the school level often include a rejection of school authority and the requirements of educational institutions, and at the community level can result in antisocial behaviors. In addition, if a child enters the education system at a disadvantage, either due to sociodemographic disadvantage or poor social inclusion, it is likely that disadvantage will carry forward throughout their schooling and impact group membership and identity at school, and subsequent academic achievement or failure (Gubbels et al., 2019). These study findings suggest connection across multiple levels is crucial for boosting levels of social inclusion and may provide opportunities to improve school completion.

### Strengths and Limitations

Strengths of the study include the longitudinal design and representative sample of youth (McMorris et al., 2007). In addition, risk and protective factors were measured by validated scales and captured across multiple levels. While this study used a strong study design, there are some limitations to consider. Firstly, all data is self-report and there are no objective measures. The gender/sex covariate may not represent the full extent of gender diversity as the measure only provided binary options. Ensuring future studies also capture gender diversity is essential (Cameron & Stinson, 2019). While the study had a representative sample stratified by suburb and school, at times replacement schools were included, which may have influenced the resulting sample distribution. As the current study examined the predictive effect of the overarching social inclusion construct on school completion, the effect of the four sub-factors was not deeply explored. Future research could examine this relationship further. Conversion of the social inclusion factor from continuous to categorical (by quartiles) may have led to some loss of nuance in the construct, and the subsequent relationship to school completion. Lastly, as this was primarily a theoretical research analysis and not validation of a scale, establishing criterion validity was not a focus and therefore the factor structure was only tested in relation to one outcome: school completion. Future research should examine this.

### Implications

These results will have application and use by school administrators, teachers, wellbeing staff, and educational psychologists. Results can be used to assist with prioritization of interventions aimed at improving school completion. Monitoring school level social inclusion and implementing programs to improve social inclusion at the school level may have positive effects on educational outcomes, and the potential to subsequently impact health and wellbeing more broadly.

Results may also be useful for governments at all levels and policy makers to increase prioritization of community level social inclusion for adolescents and to promote community connection and prosocial behavior. Adjusting existing policy and school processes to incorporate a more comprehensive model for improving social inclusion through a coordinated approach across bioecological levels may help address educational inequity and ensure maximum likelihood of school completion. Future studies using indicators specifically designed to capture the unique nature of “participation”, “access”, and “empowerment” would strengthen the operationalization of social inclusion to ensure comprehensive measurement. Modeling the change in social inclusion from the primary school years through the secondary school years to identify the impact of key transition periods may enable targeted interventions to further enhance social inclusion.

## Conclusion

Feeling included in society and having a sense of belonging within social groups is important for physical and mental health, as well as social and emotional outcomes for adolescents. The findings of this study support the notion that social inclusion has the potential to improve educational equity. Using a measure of social inclusion across two key domains and four bioecological levels, this study found social inclusion predicted Year 12 school completion in a sample of Australian youth, after accounting for other related factors. Specifically, higher social inclusion in Year 10 predicted greater odds of school completion in Year 12. Conversely, low levels of social inclusion were associated with a decreased likelihood of completing secondary school, culminating in early school leaving. An enhanced focus on promoting social inclusion in young people, prior to Year 10, may improve educational trajectories and equity among young adults, with potentially cascading benefits for adult employment pathways and quality of life.

## Supplementary information

Supplementary Material
