# Supplementary material for: Modeling Adolescent Social Inclusion to Improve School Completion
Source: J Youth Adolesc. 2023 May 29;52(8):1662–73. doi: 10.1007/s10964-023-01792-9 (PMC10276098; doi:10.1007/s10964-023-01792-9)
Supplement: Supplementary file 1 — Supplementary Material [file 10964_2023_1792_MOESM1_ESM.docx]

**Supplementary Material**

**Appendix A**

*Descriptive Statistics for Initial Group of 32 Social Inclusion Indicators at Wave 5 (N = 825)*

| Indicator | *N* | *M* | *SD* | Range |
| --- | --- | --- | --- | --- |
| Participation |  |  |  |  |
| 1. Commitment to school ^a^ | 809 | 3.80 | 0.59 | 1-5 |
| 1. Been involved in sports, clubs, activities, outside of school ^b^ | 824 | 4.21 | 2.74 | 1-8 |
| 1. Prosocial behavior ^a^ | 822 | 4.39 | 1.98 | 1-8 |
| 1. School climate - student participation ^a^ | 806 | 3.01 | 0.53 | 1-4 |
| Connectedness and belonging |  |  |  |  |
| 1. Parents tell child when they are proud of them | 818 | 2.95 | 0.89 | 1-4 |
| 1. Parents notice when child doing good job | 820 | 3.05 | 0.86 | 1-4 |
| 1. Family attachment ^a^ | 815 | 2.98 | 0.70 | 1-4 |
| 1. Feel safe at school | 806 | 3.25 | 0.69 | 1-4 |
| 1. School climate - belonging/acceptance ^a^ | 805 | 2.98 | 0.52 | 1-4 |
| 1. School climate - supportive teacher relationships ^a^ | 805 | 2.80 | 0.54 | 1-4 |
| 1. School climate – feels different to most other students ^b^ | 804 | 2.71 | 0.82 | 1-4 |
| 1. Neighbourhood attachment ^a^ | 817 | 3.09 | 0.72 | 1-4 |
| 1. Community rewards for prosocial involvement ^a^ | 819 | 2.16 | 0.88 | 1-4 |
| 1. Low level of victimization/bullying ^b^ | 822 | 3.72 | 0.54 | 1-4 |
| Citizenship and rights |  |  |  |  |
| 1. Civic engagement ^a^ | 818 | 1.69 | 0.79 | 1-5 |
| 1. Made a personal effort to care for environment | 823 | 4.06 | 2.31 | 1-8 |
| 1. Been a leader in group or organization | 818 | 1.92 | 1.22 | 1-5 |
| 1. Helped someone feel better when upset | 823 | 4.83 | 2.03 | 1-8 |
| 1. Helped student with difficulty at school | 820 | 3.88 | 1.99 | 1-8 |
| 1. Prosocial involvement: volunteering to help others ^b^ | 819 | 1.97 | 1.28 | 1-5 |
| 1. Prosocial involvement: jobs or chores in the home ^b^ | 810 | 3.05 | 0.89 | 1-4 |
| 1. Frequency of religious services or activities attendance ^b^ | 825 | 1.96 | 1.03 | 1-4 |
| Access |  |  |  |  |
| 1. Family opportunities for prosocial involvement ^a^ | 820 | 3.10 | 0.66 | 1-4 |
| 1. Lots of chances to be part of class discussion | 808 | 3.26 | 0.64 | 1-4 |
| 1. Youth groups, etc. available in neighborhood ^b^ | 816 | 3.39 | 1.21 | 1-4 |
| 1. Community service (visiting or helping people, environmental projects, fundraising for charity) available in neighborhood ^b^ | 816 | 3.26 | 1.29 | 1-4 |
| 1. Lots of adults to talk to in neighborhood | 818 | 2.25 | 0.96 | 1-4 |
| Empowerment |  |  |  |  |
| 1. Kids empowered in neighborhood (make decisions) | 815 | 2.05 | 0.89 | 1-4 |
| 1. Kids listened to in neighborhood | 815 | 2.18 | 0.93 | 1-4 |
| 1. Level of self-efficacy ^a, b^ | 816 | 3.12 | 0.51 | 1.5-4 |
| 1. Stress/Coping – demonstrates good coping ^a, b^ | 817 | 2.84 | 0.69 | 1-4 |
| 1. Demonstrates emotional control ^a, b^ | 817 | 2.71 | 0.62 | 1-4 |
| ^a^ Continuous variable. ^b^ Dropped from further analysis. | | | | |

**Appendix B**

*International Youth Development Study Youth Survey Question Items and Stems for Initial Group of 32 Indicators*

| Variable | Question | Response options |
| --- | --- | --- |
| Participation | | |
| Commitment to school ^a^ | Seven items   1. During the last four weeks how many whole days have you missed because you skipped or “cut/wagged”? | None; 1; 2; 3; 4-5; 6-10; 11 or more |
|  | 1. How often do you feel that the schoolwork you are assigned is meaningful and important? | Almost Always; Often; Sometimes; Seldom; Never |
|  | 1. How interesting are most of your school subjects to you? | Very Interesting; Quite Interesting; Fairly Interesting; Slightly Boring; Very Boring |
|  | 1. How important do you think the things you are learning in school are going to be for your later life? | Very Important; Quite Important; Fairly Important; Slightly Important; Not at all Important |
|  | *Now, thinking back over the past year in school, how often did you...*   1. Enjoy being in school? (reverse scored) 2. Hate being in school? 3. Try to do your best work in school? (reverse scored) | Never; Seldom; Sometime; Often; Almost Always |
| Been involved in sports, clubs, activities, outside of school ^b^ | How many times in the past year (12 months) have you: Been involved in sports, clubs, organizations, or other activities outside of school? | Never; 1 or 2 times; 3 to 5 times; 6 to 9 times; 10 to 19 times; 20 to 29 times; 30 to 39 times; 40+ times |
| Prosocial behavior ^a^ | Two items  How many times in the past year (12 months) have you:   1. Looked forward to going to school? 2. Been involved in sports, clubs, organizations, or other activities at school? | Never; 1 or 2 times; 3 to 5 times; 6 to 9 times; 10 to 19 times; 20 to 29 times; 30 to 39 times; 40+ times |
| School climate – student participation ^a^ | Five items   1. There are lots of chances for students at my school to get involved in sports, clubs and other activities outside class 2. At my school, students have a lot of chances to help decide and plan things like school activities, events and policies 3. Student activities at this school offer something for everyone 4. Students have a say in decisions affecting them at this school 5. Students at this school are encouraged to take part in activities, programs and special events | NO!; no; yes; YES! |
| Connectedness and sense of belonging | | |
| Parents tell child when they are proud of them | How often do your parents tell you they're proud of you for something you've done? | Never or Almost Never; Sometimes; Often; All the Time |
| Parents notice when child doing good job | My parents notice when I am doing a good job and let me know about it. | Never or Almost Never; Sometimes; Often; All the Time |
| Family attachment ^a^ | Four items   1. Do you feel very close to your mother? 2. Do you share your thoughts and feelings with your mother? 3. Do you feel very close to your father? 4. Do you share your thoughts and feelings with your father? | NO!; no; yes; YES! |
| Variable | Question | Response options |
| Feel safe at school | I feel safe at my school | NO!; no; yes; YES! |
| School climate - belonging/acceptance ^a^ | Six items   1. I can really be myself at this school 2. Other students in this school take my opinions seriously 3. Most of the students in my classes enjoy being together 4. Most of the students in my classes are kind and helpful 5. Most other students accept me as I am 6. I feel I belong at this school | NO!; no; yes; YES! |
| School climate - supportive teacher relationships ^a^ | Eight items   1. My teachers are fair in dealing with students 2. There’s at least one teacher or other adult in this school I can talk to if I have a problem 3. I feel I can go to my teacher with the things that are on my mind 4. In this school, all students’ idea are listened to and valued 5. In this school, teachers and students really trust one another 6. In this school, teachers treat students with respect 7. This school really cares about students as individual 8. Most of my teachers really listen to what I have to say | NO!; no; yes; YES! |
| School climate – feels different to most other students ^b^ | I feel very different from most other students here | NO!; no; yes; YES! |
| Neighbourhood attachment ^a^ | Three items   1. I'd like to get out of my neighborhood. 2. If I had to move, I would miss the neighborhood I now live in. (reverse scored) 3. I like my neighborhood. (reverse scored) | NO!; no; yes; YES! |
| Community rewards for prosocial behavior ^a^ | Three items   1. There are people in my neighborhood who are proud of me when I do something well. 2. There are people in my neighborhood who encourage me to do my best. 3. My neighbors notice when I am doing a good job and let me know about it. | NO!; no; yes; YES! |
| Low level of victimization/bullying ^b^ | Have you been bullied recently (teased or called names, had rumors spread about you, been deliberately left out of things, threatened physically or actually hurt)? | No; Yes, less than once a week; Yes, about once a week; Yes, most days |
| Citizenship and rights | | |
| Civic engagement ^a^ | Four items  During the past 12 months…   1. How often have you actively supported organisations that help disadvantaged people? 2. How often have you taken part in fund-raising activities (e.g., 40-Hour Famine, Walk against Want) 3. How often have you visited elderly or disabled people to cheer them up? 4. How often have you been active in my school’s organisation (e.g., SRC, class or school captain)? | Never; Once; Twice; 3-4 times; 5 or more |
| Made a personal effort to care for environment | How many times in the past year (12 months) have you made a personal effort to care for the environment in your daily life (like recycled waste or reduced energy use)? | Never; 1 or 2 times; 3 to 5 times; 6 to 9 times; 10 to 19 times; 20 to 29 times; 30 to 39 times; 40+ times |
| Been a leader in group or organization | During the past 12 months how many times have you been a leader in a group or organization in your school or in your community (for example, an “officer” or “captain”)? | Never; Once; Twice; 3-4 times; 5 or more |
| Variable | Question | Response options |
| Helped someone feel better when upset | How many times in the past year (12 months) have you helped someone feel better when they were upset? | Never; 1 or 2 times; 3 to 5 times; 6 to 9 times; 10 to 19 times; 20 to 29 times; 30 to 39 times; 40+ times |
| Helped student with difficulty at school | How many times in the past year (12 months) have you helped a student with a difficulty at school? | Never; 1 or 2 times; 3 to 5 times; 6 to 9 times; 10 to 19 times; 20 to 29 times; 30 to 39 times; 40+ times |
| Prosocial involvement: volunteering to help others ^b^ | During the past 12 months…  How often have you done volunteer work to help other people, such as helping out at a hospital or raising money for charity? | Never; Once; Twice; 3-4 times; 5 or more |
| Prosocial involvement: jobs or chores in the home ^b^ | How often do you do jobs or chores around the house? | Never; Less than once a week; Several times a week; Everyday |
| Frequency of religious services or activities attendance ^b^ | How often do you attend religious services or activities? | Never; Rarely; 1-2 Times a Month; About Once a Week or More |
| Access | | |
| Family opportunities for prosocial involvement ^a^ | Three items   1. If I had a personal problem, I could ask my mom or dad for help. 2. My parents give me lots of chances to do fun things with them. 3. My parents ask me what I think before most family decisions affecting me are made. | NO!; no; yes; YES! |
| Lots of chances to be part of class discussion | I have lots of chances to be part of class discussions or activities. | NO!; no; yes; YES! |
| Youth groups, etc. available in neighborhood ^b^ | Which of the following activities for people your age are available in your community?  Youth groups (boys and girls clubs, church groups). | Yes; No |
| Community service available in neighborhood ^b^ | Which of the following activities for people your age are available in your community?  Community service (visiting or helping people, environmental projects, fundraising for charity). | Yes; No |
| Lots of adults to talk to in neighborhood | There are lots of adults in my neighborhood I could talk to about something important. | NO!; no; yes; YES! |
| Empowerment | | |
| Kids empowered in neighborhood | In my neighborhood, kids can help decide which activities are provided and how they are run. | NO!; no; yes; YES! |
| Kids listened to in neighborhood | In my neighborhood, adults pay attention to what kids have to say. | NO!; no; yes; YES! |
| Level of self-efficacy ^a, b^ | Four items  Do you think you would handle this situation well?   1. Peer encourages you to steal a CD. 2. Mother says you cannot go out with friends on a weeknight. 3. Someone bumps into you while walking in part of town where you don’t know anyone. 4. Peer offers you a drink at a party. | NO!; no; yes; YES! |

| Variable | Question | Response options |
| --- | --- | --- |
| Stress/Coping – demonstrates good coping ^a, b^ | Two items  When I have a problem…   1. I think about the best ways to handle it. 2. I am good at working it out. | NO!; no; yes; YES! |
| Demonstrates emotional control ^a, b^ | Four items   1. I know how to relax when I feel tense. 2. I am always able to keep my feelings under control. 3. I know how to calm down when I am feeling nervous. 4. I control my temper when people are angry with me. | NO!; no; yes; YES! |
| ^a^ Continuous variable. ^b^ Dropped from further analysis. | | |

**Appendix C**

*Pearson’s Correlation Matrix for Final 21 Indicators Included in the Analysis*

|  | 1 | 2 | 3 | 4 | 5 | 6 | 7 | | | 8 | 9 | 10 | 11 | 12 | 13 | 14 | | 15 | | 16 | 17 | 18 | 19 | 20 | 21 |
| --- | --- | --- | --- | --- | --- | --- | --- | --- | --- | --- | --- | --- | --- | --- | --- | --- | --- | --- | --- | --- | --- | --- | --- | --- | --- |
| 1 | 1 |  |  |  |  |  |  | | |  |  |  |  |  |  |  | |  | |  |  |  |  |  |  |
| 2 | .46** | 1 |  |  |  |  |  | | |  |  |  |  |  |  |  | |  | |  |  |  |  |  |  |
| 3 | .40** | .39** | 1 |  |  |  |  | | |  |  |  |  |  |  |  | |  | |  |  |  |  |  |  |
| 4 | .29** | .22** | .22** | 1 |  |  |  | | |  |  |  |  |  |  |  | |  | |  |  |  |  |  |  |
| 5 | .32** | .25** | .25** | .74** | 1 |  |  | | |  |  |  |  |  |  |  | |  | |  |  |  |  |  |  |
| 6 | .27** | .20** | .18** | .54** | .53** | 1 |  | | |  |  |  |  |  |  |  | |  | |  |  |  |  |  |  |
| 7 | .33** | .26** | .37** | .11** | .14** | .15** | 1 | | |  |  |  |  |  |  |  | |  | |  |  |  |  |  |  |
| 8 | .40** | .36** | .45** | .21** | .29** | .21** | .51** | | | 1 |  |  |  |  |  |  | |  | |  |  |  |  |  |  |
| 9 | .49** | .35** | .62** | .25** | .30** | .29** | .45** | | | .57** | 1 |  |  |  |  |  | |  | |  |  |  |  |  |  |
| 10 | .26** | .23** | .23** | .21** | .20** | .28** | .28** | | | .29** | .24** | 1 |  |  |  |  | |  | |  |  |  |  |  |  |
| 11 | .19** | .24** | .26** | .25** | .26** | .25** | .16** | | | .26** | .31** | .38** | 1 |  |  |  | |  | |  |  |  |  |  |  |
| 12 | .14** | .39** | .22** | .10** | .14** | .05 | .12** | | | .13** | .15** | .16** | .24** | 1 |  |  | |  | |  |  |  |  |  |  |
| 13 | .23** | .36** | .16** | .09** | .15** | .12** | .16** | | | .15** | .15** | .13** | .05 | .31** | 1 |  | |  | |  |  |  |  |  |  |
| 14 | .14** | .37** | .14** | .10** | .14** | .08* | .08* | | | .15** | .13** | .18** | .20** | .50** | .24** | 1 | |  | |  |  |  |  |  |  |
| 15 | .15** | .38** | .21** | .10** | .10** | .04 | .12** | | | .15** | .14** | .09* | .06* | .37** | .51** | .28** | | 1 | |  |  |  |  |  |  |
| 16 | .25** | .41** | .20** | .14** | .16** | .07* | .15** | | | .21** | .23** | .11** | .13** | .37** | .50** | .32** | | .66** | | 1 |  |  |  |  |  |
| 17 | .30** | .21** | .29** | .59** | .57** | .69** | .19** | | | .27** | .37** | .32** | .32** | .09* | .13** | .12** | | .06* | | .10* | 1 |  |  |  |  |
| 18 | .34** | .27** | .53** | .15** | .17** | .09** | .41** | | | .44** | .48** | .18** | .12** | .16** | .19** | .13** | | .20** | | .21** | .21** | 1 |  |  |  |
| 19 | .18** | .20** | .26** | .24** | .23** | .26** | .13** | | | .24** | .30** | .35** | .76** | .21** | .02 | .19** | | .02 | | .07* | .32** | .10** | 1 |  |  |
| 20 | .12** | .16** | .23** | .21** | .20** | .21** | .12** | | | .22** | .25** | .30** | .75** | .18** | .06* | .16** | | .04 | | .10** | .28** | .09* | .62** | 1 |  |
| 21 | .17** | .22** | .27** | .23** | .25** | .25** | .18** | | | .29** | .32** | .36** | .75** | .19** | .05 | .15** | | .03 | | .08** | .33** | .14** | .63** | .77** | 1 |
| Key for social inclusion indicators included in matrix | | | | | | | | | | | | | | | | | | | | | | | | | |
| 1 | Commitment to school | | | | | | | 8 | School climate - belonging/acceptance | | | | | | | | 15 | | Helped someone feel better when upset | | | | | | |
| 2 | Prosocial behavior | | | | | | | 9 | School climate - supportive teacher relationships | | | | | | | | 16 | | Helped student with difficulty at school | | | | | | |
| 3 | School climate - student participation | | | | | | | 10 | Neighbourhood attachment | | | | | | | | 17 | | Family opportunities for prosocial involvement | | | | | | |
| 4 | Parents tell child when they are proud of them | | | | | | | 11 | Community rewards for prosocial behavior | | | | | | | | 18 | | Lots of chances to be part of class discussion | | | | | | |
| 5 | Parents notice when child doing good job | | | | | | | 12 | Civic engagement | | | | | | | | 19 | | Lots of adults to talk to in neighborhood | | | | | | |
| 6 | Family attachment | | | | | | | 13 | Made personal effort to care for environment | | | | | | | | 20 | | Kids empowered in neighborhood (make decisions) | | | | | | |
| 7 | Feel safe at school | | | | | | | 14 | Been a leader in group or organization | | | | | | | | 21 | | Kids listened to in neighborhood | | | | | | |
| ** p* < .05; ** *p* < .01. | | | | | | | | | | | | | | | | | | | | | | | | | |

**Appendix D**

*Questions and Response Options for Wave1 and Wave 5 Covariates*

| Variable | Question | Response options |
| --- | --- | --- |
| Demographics | | |
| Gender ^b^ | Participant gender was derived from school enrolment records. | Male; Female |
| Ethnicity | | |
| Parental ethnic background ^b^ | Of the following, what race or ethnicity do you consider yourself to be? | Australian; African; Asian; Spanish; Aboriginal; Torres Strait Islander; Pacific Islander; Mixed ethnicity (specify); Other (specify) |
|  | Collapsed into two categories - Australian ethnicity (including Aboriginal and Torres Strait Islander) or non-Australian. | Australian; Non-Australian |
| Substance use | | |
| Current alcohol use | In the past 30 days on how many occasions (if any) have you had more than just a few sips of an alcoholic beverage (like beer, wine or liquor/spirits)? | Never; 1 or 2 times; 3 to 5 times; 6 to 9 times; 10 to 19 times; 20 to 29 times; 30 to 39 times; 40+ times |
|  | Collapsed into two categories - consumed more than a few sips of alcohol in the past 30 days, or not. | No (Never); Yes (1 or more times) |
| Perceived availability of drugs in the community ^a^ | Four items   1. If you wanted to get some cigarettes, how easy would it be for you to get some? 2. If you wanted to get some alcohol (like beer, wine or liquor/spirits), how easy would it be for you to get some? 3. If you wanted to get some marijuana (pot, weed, grass), how easy would it be for you to get some? 4. If you wanted to get a drug like cocaine, heroin, LSD (acid), or amphetamines (speed), how easy would it be for you to get some? | Very hard; Sort of hard; Sort of easy; Very easy |
| Antisocial behavior | | |
| Perceived rewards for antisocial involvement ^a^ | Four items  What are the chances you would be seen as cool if you:   - 1. Smoked cigarettes?   2. Began drinking alcoholic beverages regularly, that is, at least once or twice a month?   3. Used marijuana?   4. Carried a weapon? | No or Very Little Chance; Little Chance; Some Chance; Pretty Good Chance; Very Good Chance |
| Parental attitudes favorable to antisocial behavior ^a^ | Three items  How wrong do your parents feel it would be for you to:   1. Steal something worth more than $5/10? 2. Draw graffiti, or write things or draw pictures on buildings or other property (without the owner’s permission)? 3. Pick a fight with someone? | Very wrong; Wrong; A Little Bit Wrong; Not Wrong at All. |
| Childhood emotional and behavioral problems | | |
| Childhood concentration/ attention problems ^a^ | Two items   1. I find it hard to keep concentrating on tasks 2. I get distracted easily when I’m doing work at school or other tasks. | NO!; no; yes; YES! |
| Variable | Question | Response options |
| Sensation seeking ^a^ | Three items  How many times have you:   1. Done crazy things even if they are a little dangerous. 2. Done something dangerous because someone dared you to do it. 3. Done what feels good no matter what. | Never; I’ve done it, but not in the past year; Less than once a month; About once a month; 2 or 3 times a month; Once a week or more. |
| Parenting problems | | |
| Parental overcontrol ^a^ | Two items   1. My parents try to control everything. 2. My parents treat me like a baby and try to protect me from everything. | NO!; no; yes; YES! |
| Mental health problems | | |
| Depression symptomology ^a^ | Thirteen items  Please indicate how true each of the following statements has been for you during the last 30 days (1 month):   1. I felt miserable or unhappy. 2. I didn’t enjoy anything at all. 3. I felt so tired I just sat around and did nothing. 4. I was very restless. 5. I felt I was no good anymore. 6. I cried a lot. 7. I found it hard to think properly or concentrate. 8. I hated myself. 9. I was a bad person. 10. I felt lonely. 11. I thought nobody really loved me. 12. I thought I could never be as good as other kids. 13. I did everything wrong. | True; Sometimes True; Not True |
| ^a^ Continuous variable. ^b^ Wave 1 covariate. All other covariates were measured in wave 5. | | |

**Appendix E**

*Exploratory Factor Analysis Factor Structure of 4-Factor Social Inclusion Model and Factor Loadings (*N *= 825)*

| Indicator | Factor loading | | | | Sum of *R*^2^ |
| --- | --- | --- | --- | --- | --- |
|  | 1 | 2 | 3 | 4 |  |
| Peer-Individual Level | | | | | |
| Helped someone feel better when upset | **.83** |  |  |  | 0.76 |
| Helped student with difficulty at school | **.81** |  |  |  | 0.78 |
| Made a personal effort to care for environment | **.65** |  |  |  | 0.55 |
| Prosocial behavior | **.56** |  |  | .50 | 0.72 |
| Civic engagement | **.54** |  |  |  | 0.42 |
| Been leader in group or organization | **.51** |  |  |  | 0.41 |
| Community Level | | | | | |
| Kids listened to in neighborhood |  | **.92** | .33 | .37 | 1.11 |
| Kids empowered in neighborhood |  | **.90** |  |  | 0.98 |
| Community rewards for prosocial |  | **.89** | .33 | .31 | 1.01 |
| Lots of adults to talk to in neighborhood |  | **.79** | .35 | .33 | 0.76 |
| Neighbourhood attachment |  | **.41** | .30 | .36 | 0.41 |
| Family Level | | | | | |
| Parents tell child when they are proud of them |  |  | **.91** |  | 1.03 |
| Parents notice when child doing good job |  | .30 | **.90** | .35 | 1.08 |
| Family opportunities for prosocial involvement |  | .38 | **.72** | .39 | 0.82 |
| Family attachment |  |  | **.68** | .30 | 0.63 |
| School Level | | | | | |
| School climate - supportive teacher relationships |  | .34 | .37 | **.79** | 0.92 |
| Lots of chances to be part of class discussion | .30 |  |  | **.71** | 0.66 |
| School climate - belonging/acceptance |  | .30 | .30 | **.71** | 0.73 |
| School climate - student participation |  | .30 |  | **.71** | 0.75 |
| Feel safe at school |  |  |  | **.68** | 0.59 |
| Commitment to school | .30 |  | .38 | **.59** | 0.62 |
| No. items per factor | 6 | 5 | 4 | 6 |  |
| Goodness of fit |  |  |  | Estimates | |
| Comparative fit index (CFI) |  |  |  | .95 | |
| Tucker-Lewis index (TLI) |  |  |  | .92 | |
| Standardized root mean square residual (SRMR) |  |  |  | .04 | |
| Root mean square error of approximation (RMSEA) |  |  |  | .07 | |
| *Note.* 1, 2, 3, and 4 in the column headings refer to Citizenship, Connectedness to Community, Connectedness to Family, and Connectedness to School, respectively. Factor loadings are standardized. Factor loadings < .30 are not displayed as they are a poor measure of the construct (Hair et al., 2014). Values in bold highlight the highest factor loadings for each item and hence the factor structure.  Sum of *R^2^* = summation of the squared standardized loading for each factor. | | | | | |

**Appendix F**

*Exploratory Factor Analysis Factor Structure of 6-Factor Social Inclusion Model and Factor Loadings (*N *= 825)*

| Indicator | Factor loading | | | | | | Sum of *R*^2^ |
| --- | --- | --- | --- | --- | --- | --- | --- |
|  | 1 | 2 | 3 | 4 | 5 | 6 |  |
| Peer-Individual Level | | | | | | | |
| Helped someone feel better when upset | **.86** |  | .44 |  |  |  | 0.27 |
| Helped student with difficulty at school | **.80** |  | .48 | .31 |  |  | 0.36 |
| Made a personal effort to care for environment | **.67** |  | .39 |  |  |  | 0.28 |
| Been leader in group or organization | .40 |  | **.75** |  |  |  | 0.64 |
| Civic engagement | .46 |  | **.67** |  |  |  | 0.51 |
| Prosocial behavior | .49 |  | **.57** | .51 |  |  | 0.68 |
| Community Level | | | | | | | |
| Kids listened to in neighborhood |  | **.92** |  | .36 | .30 |  | 0.35 |
| Kids empowered in neighborhood |  | **.91** |  |  |  |  | 0.23 |
| Community rewards for prosocial |  | **.88** | .35 | .30 | .30 |  | 0.35 |
| Lots of adults to talk to in neighborhood |  | **.79** | .34 | .32 | .32 |  | 0.38 |
| Neighbourhood attachment |  | **.39** |  | .35 |  | .33 | 0.36 |
| Family Level | | | | | | | |
| Parents tell child when they are proud of them |  |  |  | .30 | **.94** |  | 1.09 |
| Parents notice when child doing good job |  | .30 |  | .37 | **.89** | .31 | 1.08 |
| Family opportunities for prosocial involvement |  | .37 |  | .39 | **.67** | .61 | 1.00 |
| Family attachment |  |  |  |  | .62 | **.72** | 1.00 |
| School Level | | | | | | | |
| School climate - supportive teacher relationships |  | .34 |  | **.79** | .33 |  | 0.84 |
| Lots of chances to be part of class discussion |  |  |  | **.72** |  |  | 0.61 |
| School climate - student participation |  |  |  | **.72** | .33 |  | 0.68 |
| School climate - belonging/acceptance |  | .30 |  | **.71** |  |  | 0.68 |
| Feel safe at school |  |  |  | **.68** |  | .30 | 0.60 |
| Commitment to school |  |  |  | **.59** | .35 |  | 0.60 |
| No. items per factor | 3 | 5 | 3 | 6 | 3 | 1 |  |
| Goodness of fit |  |  |  |  |  | Estimates | |
| Comparative fit index (CFI) |  |  |  |  |  | .98 | |
| Tucker-Lewis index (TLI) |  |  |  |  |  | .95 | |
| Standardized root mean square residual (SRMR) |  |  |  |  |  | .02 | |
| Root mean square error of approximation (RMSEA) |  |  |  |  |  | .06 | |
| *Note.* 1 to 6 in the column headings refer to each of the factors in the 6-factor structure. Factor loadings are standardized. Factor loadings < .30 are not displayed as they are a poor measure of the construct (Hair et al., 2014). Values in bold highlight the highest factor loadings for each item and hence the factor structure.  Sum of *R^2^* = the summation of the squared standardized loading for each factor. | | | | | | | |

**Appendix G**

*Sensitivity Analyses for Social Inclusion Measures – Logistic Regression to Determine Missingness*

| Variable | Odds Ratio | Standard Error | 95% CI | |
| --- | --- | --- | --- | --- |
|  |  |  | *LL* | *UL* |
| Social inclusion |  |  |  |  |
| Second quartile | 0.85 | 0.30 | 0.43 | 1.71 |
| Third quartile | 0.75 | 0.28 | 0.36 | 1.56 |
| Fourth quartile (highest level) | 1.07 | 0.40 | 0.51 | 2.25 |
| Covariates |  |  |  |  |
| Gender – female | 0.37*** | 0.10 | 0.22 | 0.63 |
| Parental ethnic background – non-Australian | 0.94 | 0.31 | 0.50 | 1.76 |
| Current alcohol use (past 30 days) | 0.78 | 0.22 | 0.45 | 1.35 |
| Perceived availability of drugs in community | 1.30 | 0.22 | 0.93 | 1.80 |
| Perceived rewards for antisocial involvement | 0.80 | 0.12 | 0.60 | 1.07 |
| Parental attitudes favorable toward antisocial behavior | 1.24 | 0.31 | 0.76 | 2.02 |
| Childhood behavior problems, concentration/attention | 1.44* | 0.25 | 1.03 | 2.02 |
| Sensation seeking | 1.05 | 0.10 | 0.86 | 1.28 |
| Parental overcontrol | 1.29 | 0.20 | 0.95 | 1.74 |
| Depression symptomology | 0.80 | 0.22 | 0.46 | 1.37 |
| CI = confidence interval; *LL* = lower limit; *UL* = upper limit.  * *p* < .05; *** *p* < .001. | | | | |
